# Evaluation of the mechanism and principles of management of temporomandibular joint dislocation. Systematic review of literature and a proposed new classification of temporomandibular joint dislocation

**DOI:** 10.1186/1746-160X-7-10

**Published:** 2011-06-15

**Authors:** Babatunde O Akinbami

**Affiliations:** 1 University of Port Harcourt Teaching Hospital, Port Harcourt, Rivers State, Nigeria

## Abstract

**Background:**

Virtually all the articles in literature addressed only a specific type of dislocation. The aim of this review was to project a comprehensive understanding of the pathologic processes and management of all types of dislodgement of the head of the mandibular condyle from its normal position in the glenoid fossa. In addition, a new classification of temporomandibular joint dislocation was also proposed.

**Method and materials:**

A thorough computer literature search was done using the Medline, Cochrane library and Embase database. Key words like *temporo-mandibular joint dislocation *were used for the search. Additional manual search was done by going through published home-based and foreign articles. Case reports/series, and original articles that documented the type of dislocation, number of cases treated in the series and original articles. Treatment done and outcome of treatment were included in the study.

**Result:**

A total of 128 articles were reviewed out which 79 were found relevant. Of these, 26 were case reports, 17 were case series and 36 were original articles. 79 cases were acute dislocations, 35 cases were chronic protracted TMJ dislocations and 311 cases were chronic recurrent TMJ dislocations. Etiology was predominantly trauma in 60% of cases and other causes contributed about 40%. Of all the cases reviewed, only 4 were unilateral dislocation. Various treatment modalities are outlined in this report as indicated for each type of dislocation.

**Conclusion:**

The more complex and invasive method of treatment may not necessarily offer the best option and outcome of treatment, therefore conservative approaches should be exhausted and utilized appropriately before adopting the more invasive surgical techniques.

## Introduction

The mechanism of temporomandibular joint dislocation varies depending on the type of dislocation which may be acute, chronic protracted or chronic recurrent dislocation [1]. This mechanics is closely related to the structure and function of the temporomandibular joint as well as the dynamics of the masticatory system [1]. The capsule of the joint is the most important structure which stabilizes the joint reinforced by the lateral ligaments [2,3]. However displacement of the head of the condyle out of the glenoid fossa is also greatly influenced by the morphology of the condyle, glenoid fossa, articular eminence, zygomatic arch and squamotympanic fissure [1,3-5]. The afore-mentioned factors mainly determine the type and direction of dislocation. In addition, age, dentition, cause and duration of the dislocation as well as the function of the masticatory muscles contribute significantly in the mechanism and management of temporomandibular joint dislocation [4-7]. The outcome of T.M.J dislocation, especially the chronic recurrent and chronic protracted are not very predictable and this depend on thorough evaluation, treatment planning and compliance of the patient [3-9].

The aim of this article is to review, analyze and update the mechanisms and various management options available for the different types of T.M.J dislocation in literature.

## Methods and materials

A thorough computer literature search was done using the Medline, Cochrane library and Embase database. Key words like *temporo-mandibular joint dislocation *were used for the search. Additional manual search was done by going through published home-based and foreign articles. Relevance of article for inclusion into the study was assessed based on the title, abstract and contents of the full article. The reference list of the reviewed articles was also searched from Medline and Embase database as well as the Thomson Reuters, Wolters Kluwer, Lippincott Williams and Wilkins search engines for Cited and Related literature search based on the relevant title. Case reports/series, and original articles that documented the type of dislocation, number of cases treated in the series and original articles. Treatment done and outcome of treatment were included in the study. Total number of each type of dislocation reported was recorded and the treatment options for each type was analyzed based on the clinical and radiological outcome (presence/absence of pain, occlusion derangement, inter-incisal distance, deviation of the mandible and masticatory function, as well as position of the condylar head in the glenoid fossa). A new classification was proposed for Temporomandibular joint dislocation based on the position of the condylar head in relation to the articular eminence.

## Results

A total of 128 articles were reviewed out which 79 were found relevant. Of these, 26 were case reports, 17 were case series and 36 were original articles. 79 cases were acute dislocations, 35 cases were chronic longstanding TMJ dislocations and 311 cases were chronic recurrent TMJ dislocations(Table 1). Etiology was predominantly trauma in 60% of cases and this include fall, road traffic accident, domestic accidents, interpersonal violence; other causes like excessive mouth opening from yawning, laughing, singing, prolonged mouth opening from oral and ENT procedures, forceful mouth opening from anaesthetic and endoscopic procedures contributed about 40%.

**Table 1:** Distribution of the articles reviewed and type of dislocation

Parameter	Frequency
***No. of articles reviewed***	*128*
***No. of relevant articles***	*79*
***Type of articles***	
*Case reports*	*26*
*Case series*	*17*
*Original articles*	*36*
***Type of dislocation***	
*Acute*	*79*
*Chronic*	*35*
*Recurrent*	*311*
*Superior*	*8*
*Medial*	*11*
*Lateral*	*2*
*Bilateral*	*421*
*Unilateral*	*4*

Of all the cases reviewed, only 4 were unilateral dislocation. Prognatism of the lower jaw, anterior cross bite and open bite were the classical features in the bilateral cases while deviation of the mandible, shift in the midline to the unaffected side and cross bite on that side were predominant in the unilateral cases.

Acute dislocation was treated by manual reduction in 63 cases without any form of anaesthesia while 2 cases were treated under IV analgesia and sedatives, and manual reduction was achieved in 14 cases under general anaesthesia, 3 cases were reduced with gag reflex (Table 2).

**Table 2:** Treatments useful for Acute dislocation

Treatment	No. of patients
*Manual reduction without anaesthesia*	*63*
*Manual reduction with Local Anaesthesia (L.A)+ sedation*	*2*
*Reduction with gag reflex*	*3*
*Manual reduction with General Anaesthesia (GA)*	*13*
*Assisted (open) reduction with General Anaesthesia*	*1*

Chronic protracted TMJ dislocations were treated by manual Hippocratic method under G.A in 13 cases, a hook was used to apply traction on the sigmoid notch in 1 case, external elastic traction with arch bars and elastic bands was used in 1 case while assisted open reduction with Bristow's elevator was done in one case (Table 3 ). Vertical-oblique ramus osteotomy was used to correct prognatism and anterior open/cross bite in 9 cases, inverted L osteotomy was used in 1 case and sagittal split osteotomy was used in 1 case (Table 3**contd**).

**Table 3:** Treatments Useful for Chronic protracted dislocation

Conservative Treatment of Chronic protracted dislocation	No. of patients
*Manual reduction without anaesthesia*	*0*
*Manual reduction with Local Anaesthesia (L.A)+ sedation*	*1*
*Manual reduction with L.A +sedation + nerve block*	*1*
*Manual reduction with General Anaesthesia (GA)*	*13*
*Assisted (open) reduction with General Anaesthesia*	*1*
*Elastic traction with Intermaxillary fixation*	*1*
*Traction with bone hook*	*1*
*Mandibular guidance therapy*	*1*
**To reposition condyle in fossa *(There was much restriction of movement)***	
*Temporalis Myotomy*	*4*
*High Condylotomy*	*?*
**To correct fusion and restore the joint (*There was complete restriction of movement*)**	
*Low Condylotomy*	*?*
*Condylectomy (for chronic lateral dislocation)*	*2*
*Gap Arthroplasty (for superior dislocation)*	*8*
**To maintain new joint and correct occlusion *(There was little restriction of movement)***	
*Horizontal subsigmoid osteotomy*	*?*
*Vertical/Oblique Osteotomy*	*9*
*Inverted L Shaped Osteotomy*	*1*
*Bilateral Sagittal Split Osteotomy*	*1*
*Midline Mandibulotomy*	*1*

The number of cases treated by chemical capsuloraphy using sclerosing agents and platelet rich plasma could not be ascertained, 9 cases were treated with autologous blood in the superior joint space and 9 cases with injection into the space and pericapsular tissues. Also, number of cases of chronic recurrent dislocation that received surgical capsuloraphy and placation for lax capsule and ligaments was not obtained. Closed low level condylotomy to allow free movement was done in 2 cases while 4 cases received open condylotomy. Fifteen cases were treated with lateral pterygoid myotomy via intraoral approach (Table 4**contd**).

**Table 4:** Treatments useful for Chronic Recurrent Dislocation

*Conservative Treatment of Recurrent dislocation*	*No. of patients*
*Chemical Capsuloraphy*	*?*
*Alcohol*	
*Rivanol(aethedicaine)*	
*Sodium monoruate*	
*Sodium psyliate (Sylnasol)*	
*Sodium tetradecyl sulphate*	
*Autologous blood capsuloraphy*	*18*
*Platelet rich plasma capsuloraphy*	*?*
*Botulinium toxin A toxin muscle injection*	*2*
**To restrict condylar movement**	
*Surgical capsuloraphy/Restitution of ligaments*	*?*
*Use of Mersilene or fascia lata around condylar neck*	*?*
*Internal Lateral pterygoid myotomy*	*15*
*External Lateral pterygoid myotomy*	*1*
*Open Condylotomy*	*4*
*Closed Condylotomy*	*2*
**To recreate mechanical obstruction along condylar path**	
*Anterior reposition/forward placement of disc*	*?*
*Downward and inward fracture of zygomatic bone*	*?*
*Eminoplasty with screws or L shaped pins*	*3*
*Eminoplasty with plates, mini-anchors*	*24*
*Eminoplasty with inlay bone grafts (Norman procedure)*	*4*
*Eminoplasty with onlay bone gafts (Dautery procedure)*	*60*
**To remove mechanical obstacle along condylar path**	
*Eminectomy (Standard)*	*95*
*Eminectomy (Modified)*	*1*
*Arthroscopic eminectomy*	*5*

? (Questionable)-signify that the number of cases treated by the treatment method could not be obtained.

Also, 101 cases of chronic recurrent dislocation were treated with eminectomy to shorten the articular eminence and allow unrestricted movement/spontaneous reduction of the condylar head. Eminoplasty using miniplates was done in 24 patients and screws was used in 3 cases (Table 4**contd**).

There were problems of severe pain and resorption of condylar head and eminence in 20% of cases with screws. Augmentation of the height of eminence by eminoplasty using bone grafts was done on some patients, 4 patients received interpositional (inlay) eminoplasty without need for wiring or plating while 60 received eminoplasty with onlay grafts wired to the articular eminence. Modified mini-invasive eminectomy and relocation of the lateral pterygoid muscle was done in 1 case.

Similar procedure was done for both sides in each bilateral case of chronic protracted and recurrent dislocation. Majority of the cases were adequately followed up for a period of 2-5 years and complications were mainly found in the patients that did eminoplasty with screws. Patients with autologous blood injected around the pericapsular tissues and into the superior joint space had less recurrence rate than those injected into the space alone.

## Discussion

### Aetio-pathogenesis of Temporomandibular Joint Dislocation

Dislocation of the temporomandibular joint is the dislodgement of the head of the condyle from its normal position in the glenoid fossa located in the squamo-temporal portion of the cranial base. It can be partial (subluxation) or complete (luxation), bilateral or unilateral, acute, chronic protracted or chronic recurrent [7-29]. Also, it can be anterior-medial, superior, medial, lateral or posterior dislocation and the cause is either spontaneous or induced by trauma, [30-80] forceful mouth opening from endotracheal intubation with laryngeal mask or tracheal tube, ENT/Dental procedures, endoscopy, excessive mouth opening from yawning, laughing, vomiting and also during seizures [81-84,94,96-111]. Altered structural components include a lax capsule, weak ligaments, small/short and atrophic condyle, atrophic articular eminence, elongated articular eminence, hypoplastic zygomatic arch and small, poorly grooved glenoid fossa. Predisposing factors include epilepsy, severe vomiting, Ehlers-Danlos syndrome and Marfan's syndrome and dystonic movements from the effect of major tranquilizers/neuroleptics used for neuro-psychiatric diseases [5,28,46,51,68,116].

Anterior dislocations are the most common and occur due to displacement of the condyle anterior to the articular eminence of the temporal bone. Anterior dislocations are usually secondary to an interruption in the normal sequence of muscle action when the mouth closes from extreme opening [30]. The masseter and temporalis muscles elevate the mandible before the lateral pterygoid muscle relaxes resulting in the mandibular condyle being pulled out of the glenoid fossa and anterior to the bony eminence. Spasm of the masseter, temporalis and pterygoid muscles causes trismus and keeps the condyle from returning into the glenoid fossa [30].

Posterior dislocations typically occur secondary to a direct blow to the chin. The mandibular condyle is pushed posteriorly toward the mastoid [34]. Injury to the external auditory canal from the condylar head may occur from this type of injury.

Superior dislocations, also referred to as central dislocations, can occur from a direct blow to a partially opened mouth. The angle of the mandible in this position and small rounded margined condyle head predisposes to upward migration of the condyle. This can result in fracture of the glenoid fossa with mandibular condyle dislocation into the middle skull base [26,35,52,109]. Further injuries from this type of dislocation can range from facial nerve injury, to intracranial hematomas, cerebral contusion, leakage of cerebrospinal fluid, and damage to the eighth cranial nerve resulting in deafness. Medial dislocations are second to anterior dislocations. Avrahami et al. [110] documented 11 cases of medial dislocation and stated that they occur due to the sustained pull of the lateral pterygoid muscle on the condyle of the affected side.

Lateral dislocation is usually associated with mandible fractures. It could be type I (subluxation) or type II (luxation). Type II has been further sub-classified into three, depending on the duration and management applicable. The condylar head migrates laterally and superiorly and can often be palpated in the temporal space [8,9,61].

Acute dislocation presents within 2 weeks and it is readily reducible by the Hippocratic maneuver. After 2 weeks, spasms and shortening of the temporalis and masseter muscles occur and reduction becomes difficult to achieve manually. This leads to the commencement of chronic protracted dislocation. Also, elongated articular eminence may prevent the sliding back of the head of the condyle into the normal position in the glenoid fossa, in this case, a chronic protracted dislocation with formation of a new pseudojoint with varying degree of movement sets in and such patients have problems with difficulty in closing the mouth (open lock) and deranged occlusion in which there is prognathism of the mandible with anterior cross bite [70].

In cases due to trauma, fibrous and bony consolidation occur anterior to the articular eminence following the sustained pull of lateral pterygoid and head of condyle resulting in ankylosis and deranged occlusion [31,34,63].

Chronic recurrent dislocations which include those due to habitual wide mouth opening are usually spontaneous and self reducible depending on the degree of alteration of the morphology of the temporomandibular joint and the contiguous structures. When the articular eminence is long, the dislocation will not be easily self reducible. It occurs commonly in patients with hypoplastic eminence, narrow fossa, lax capsule, collagen disorders, small condyle, hypermobility syndromes, oromandibular dystonias and use of neuroleptic drugs [17,18]

Plain T.M.J views especially the transcranio-oblique views, plain and contrast CT scans, i-CAT scans and MRI, linear and rotational plain digital tomograms, are joint arthroscopy are useful to assess the position of the head of the condyle and meniscus in relation to the glenoid fossa, mastoid process, tympanic plate and articular eminence. Recent imaging tools include the Dolphin imaging system which imports 2D facial photographs (facial wrap) on 3D stereographic images to enhance treatment simulation [40,41,55].

### Newly Proposed Classification of TMJ Dislocation based on the position of the head of the condyle to the articular eminence

The author has classified dislocation based on relationship of the head of mandibular condyle to the articular eminence seen on clinico-radiological evaluation into three types (I-III).

***Type I*** - *the head of condyle is directly below the tip of the eminence*

***Type II*** - *the head of condyle is in front of the tip of the eminence*

***Type III*** - *the head of condyle is high up in front of the base of the eminence*.

### Conservative and Surgical Interventions in TMJ Dislocations

Cases of acute antero-medial, medial, lateral, or posterior dislocation can be reduced manually under local or general anaesthesia, analgesic control with or without sedation. Other methods that have been used in literature include the wrist-pivot technique by Lowery et al. 2004, [30] combined ipsilateral staggering technique by Thomas et al. 2006, [97] the extraoral technique by Chen et al. 2007 [43]. Awang et al.1987 [71] also described the gag reflex procedure in which the soft palate is rubbed across with a dental probe to initiate relaxation of the lateral pterygoid muscle and spontaneous reduction and closure of the mouth. However, Hippocratic maneuver still has the highest success rate.

Acute or chronic superior dislocation is better treated by gap arthroplasty, the impacted condylar head in the middle cranial fossa must be left in place to avoid bleeding, cerebrospinal fluid leakage and infection [26,35,52,64].

Condylectomy is done for prolonged type II (complete/luxation) lateral dislocations, while treatment of type I and early type II is achieved by closed reduction of both dislocation and associated mandibular fractures, and intermaxillary fixation using arch bars, stainless steel ligature and tie wires for 4-6 weeks [9,61]. When there is bilateral condylar extracapsular fracture, open reduction and internal fixation of the condylar segment is indicated in addition to intermaxillary fixation with elastic bands for 2-4 weeks [8,9,61].

For chronic protracted dislocation, it is usually in the proposed type III position, the Hippocratic maneuver is usually unsuccessful without general anaesthesia and muscle relaxants and even with that, failure rate is high. Conservative methods like elastic rubber traction with arch bars and ligature wires/IMF with elastic bands are useful to achieve reduction in chronic protracted dislocation. Prior to the use of elastic bands, acrylic blocks or impression compound spacer can be placed in between upper and lower teeth to depress the mandible and open up the bite posteriorly, this helps displace the condyle downwards, the elastic bands that are applied front backwards helps to push the mandible/condyle backwards into the fossa after removing the spacer in about 72 hrs to 1 week. Extrusion of the teeth has been reported and it is corrected with bite plane [15,103].

Bone hook has also been used to apply traction via the sigmoid notch [15] Traction with wires is also possible through holes drilled in the angle of the mandible [15].

Manual reduction under local anaesthesia/deep temporal and masseteric nerve blocks [83], conscious sedation and general anesthesia or have also been described and these should be done first, but most cases are mainly amenable to surgical procedures.

In 1981, Lewis et al. [84] used Bristow's elevator to push the condyle into position via the Gillie's temporal approach for zygomatic bone elevation, the incision was extended to the preauricular region. This is the assisted reduction under general anaesthesia.

When surgery is indicated for chronic protracted/prolonged dislocation (CPD) especially cases with longer duration, the goal may be to reposition the condyle in the glenoid fossa and restore movement; or if there is movement in the new position, the goal will be to set back the protruded mandible and correct occlusion. When temporalis is short and spastic, Laskin proposed an intraoral surgical approach to the muscle via a coronoid incision to do a temporalis myotomy [100]. Where access is difficult, when there is fibrosis or adhesions of muscle and cases where reunion of the muscles may occur, coronoidotomy with or without condylotomy is advocated [15].

Gotlieb advocated condylectomy and coronoidectomy in cases where there is ankylosis but there is possibility of entering the base of the skull and excessive bleeding from pterygoid plexus, internal maxillary and middle meningeal vessels [112].

Vertical and oblique ramus osteotomies have therefore, been described by many authors but these have the disadvantages of less bone contact, and impingement/impaction of the coronoid process on the condyle and the new joint, causing restrictions of movement [20,23,77,112,113].

Inverted L-shaped ramus osteotomy has been described by Adekeye in 1976 [112] to ensure maximal bone contact which is necessary for stability and healing.

Bilateral sagittal split technique offers a better outcome for correction of occlusion because there is no extraoral scar, risk of damage to inferior alveolar bundle is reduced in experienced hands and there is improved bone contact. Also, there is less post-operative bleeding and swelling from muscular detachment [20,112].

Chronic recurrent dislocation can be approached by conservative procedures of injecting sclerosing agents, autologous blood or platelet rich plasma into the lax pericapsular tissue and superior joint space weekly over a period of 6 weeks, [11-13,65,113] the sclerosing agents include sodium psyliate or sodium tetradecyl sulphate. Other workers have injected steroids into and around the capsule. The action of these injections is to induce fibrosis.

Moore and Wood [73,99] as well as some other workers have also injected Botulinium A toxin into the lateral pterygoid muscle, it is a protein catalyst, which prevents release of acetycholine at the neuromuscular junction. It reversibly inactivate the protein that binds synaptic vesicles with the cell membrane. This is particularly useful when chronic recurrent dislocation is due to tardive dyskinesia and dystonias. It causes dysarthria, nasal speech, nasal regurgitation, painful chewing and swallowing, myasthenia gravis-like syndrome when it spreads to adjacent tissues and it is contraindicated in pregnancy and lactating mothers.

Chronic recurrent dislocations not due to oromandibular dystonia respond better to surgical approaches especially when chemical capsuloraphy fails. These include surgical capsuloraphy in which a wedge of the capsule is excised and the tissue repaired (capsular placation) with the goal of restituting and reinforcing the lax capsule, in situations where the eminence is low, it can be augmented or reconstructed with screws, plates or implants to improve the height.

Depending on the predisposing factor or morphology of the temporomandibular joint, the surgical procedures used to treat chronic recurrent dislocation may be directed towards restricting the condylar movement, creating a mechanical obstacle along the path of condylar translation or removing the mechanical obstacle in the condylar path.

Many surgeons have enhanced self reduction of chronic recurrent dislocation that cannot be easily self reduced due to elongated articular eminence by performing total eminectomy described by Myraugh in 1951 [104] to remove the eminence which serve as an obstruction [1,4,27,66,69,79,104,105,107]. Others have utilized the concept of restriction of condylar movement by detachment of lateral pterygoid tendon via internal or external approach or by doing an open or closed condylotomy below the attachment of lateral pterygoid [17,24,25,59,60,87,100]. Closed surgery reduces scarring but access is poor and increased damage to nerves and vessels in unskilled hands. The chance of recurrence due to fibrosis or re-union after surgery is higher for myotomy than condylotomy. Fascia lata, Mersilene tapes (Dacron) anchored around the zygomatic arch and passed around the condyle have also been used to restrict its movement [103]. Fascia lata can be readily harvested and treatment is cheaper but accompanied with post-operative pain, swelling, minor gait disturbance and movement of the lower limb [103].

Dautery and his colleagues in other to create mechanical obstacle along the condylar path, performed an osteotomy of the zygomatic arch and displaced the anterior segment downwards and inward to serve as a stop to the forward and upward movement of the condyle head [103]. This cannot be done in elderly patients because of the brittleness of the bone. Other materials like silicone wedge blocks and coralline hydroxyappatite blocks have been used [114]. Looseness, displacement and immune reactions are some of the adverse effects, especially for silicones.

Norman and his colleagues used interpositional eminoplasty in which bone grafts from the iliac or calvaria was sandwiched in the gap created in the middle of the articular eminence [5]. They did not use wires or plates fix the segments. On the other hand, Dautery also performed an onlay eminoplasty by adapting a graft (fixed with wires) directly on the eminence to increase the height [78]. Both procedures also served to create mechanical obstruction. The grafts must extend medially to prevent medial escape.

Although procedures on the articular eminence seems to be gaining much popularity in recent times, eminectomy whose validity has been demonstrated by several authors, removes the bony obstacle, preventing condylar locking, but, these procedures does not address the un-coordinated muscle activity and the lax capsule or ligament, and this made some surgeons to adopt a modified mini-invasive eminectomy and relocation of the lateral pterygoid muscle or redirection of the temporalis muscle which aims to act on both the obstacle and the cause with respect to restoration of TMJ biomechanical constraints [1,75,103,115]. However, about 95% success rate have been recorded after use of eminectomy and metallic implants on the articular eminence.

Meniscoplasties and menisectomies are relevant procedures done when altered disc morphology and position cause dislocation or prevent self reduction. Total joint replacements should be considered when all appropriate treatments fail in chronic protracted and chronic recurrent dislocations, especially those with associated degenerative joint diseases [4,14,18,28,103,107].

## Conclusion

The difficulty index assesses the ease of the manual reduction method to achieve a reduction of the condyle into its normal position in acute or chronic protracted dislocation and it is directly related to the position of the condylar head and the height of the articular eminence. Also, the frequency of recurrent dislocation and the self reducibility can be inversely linked to the height of the articular eminence.

The more complex and invasive method of treatment may not necessarily offer the best option and outcome of treatment, therefore conservative approaches should be exhausted and utilized appropriately before adopting the more invasive surgical techniques which should be done after thorough assessment and treatment planning [103]. Surgical treatment must therefore be based on the type, mechanism, aetio-pathogenesis and predisposing factor/morphology of the joint, age, availability of materials and skilled manpower.

## Competing interests

The author declares that they have no competing interests.

## References

[B1] Gay-Escoda C Eminectomy associated with redirectioning of the temporal muscle for treatment of recurrent TMJ dislocation J Craniomaxillofac Surg 1987 15 6 355 8 3480900 10.1016/s1010-5182(87)80082-3

[B2] Dimitroulis G The use of dermis grafts after discectomy for internal derangement of the temporomandibular joint J Oral Maxillofac Surg 2005 63 2 173 8 10.1016/j.joms.2004.06.051 15690284

[B3] Güven O Review Inappropriate treatments in temporomandibular joint chronic recurrent dislocation: presenting three particular cases J Craniofac Surg 2005 16 3 449 52 10.1097/01.SCS.0000147389.06617.F8 15915113

[B4] Vasconcelos BC Porto GG Neto JP Vasconcelos CF Review Treatment of chronic mandibular dislocations by eminectomy: follow-up of 10 cases and literature review Med Oral Patol Oral Cir Bucal 2009 14 11 e593 6 19680206 10.4317/medoral.14.e593

[B5] Medra AM Mahrous AM Glenotemporal osteotomy and bone grafting in the management of chronic recurrent dislocation and hypermobility of the temporomandibular joint Br J Oral Maxillofac Surg 2008 46 2 119 22 10.1016/j.bjoms.2007.08.004 17935842

[B6] He D Yang C Chen M Jiang B Wang B Intracapsular condylar fracture of the mandible: our classification and open treatment experience J Oral Maxillofac Surg 2009 67 8 1672 9 10.1016/j.joms.2009.02.012 19615581

[B7] Gao C Long-term results of the treatment of mandibular condyle fracture Zhonghua Kou Qiang Yi Xue Za Zhi 1989 24 4 228 9 255 2517099

[B8] Landes CA Lipphardt R Prospective evaluation of a pragmatic treatment rationale: open reduction and internal fixation of displaced and dislocated condyle and condylar head fractures and closed reduction of non-displaced, non-dislocated fractures. Part I: condyle and subcondylar fractures Int J Oral Maxillofac Surg 2005 34 8 859 70 10.1016/j.ijom.2005.04.021 15979851

[B9] Ferguson JW Stewart IA Whitley BD Review Lateral displacement of the intact mandibular condyle. Review of literature and report of case with associated facial nerve palsy J Craniomaxillofac Surg 1989 17 3 125 7 2651482 10.1016/s1010-5182(89)80084-8

[B10] Zachariades N Mezitis M Mourouzis C Papadakis D Spanou A Review Fractures of the mandibular condyle: a review of 466 cases. Literature review, reflections on treatment and proposals J Craniomaxillofac Surg 2006 34 7 421 32 17055280 10.1016/j.jcms.2006.07.854

[B11] Machon V Abramowicz S Paska J Dolwick MF Autologous blood injection for the treatment of chronic recurrent temporomandibular joint dislocation J Oral Maxillofac Surg 67 1 114 9 10.1016/j.joms.2008.08.044 19070756

[B12] Hasson O Nahlieli O Autologous blood injection for treatment of recurrent temporomandibular joint dislocation Oral Surg Oral Med Oral Pathol Oral Radiol Endod 2001 92 4 390 3 10.1067/moe.2001.116602 11598572

[B13] Pinto AS McVeigh KP Bainton R The use of autologous blood and adjunctive 'face lift' bandage in the management of recurrent TMJ dislocation Br J Oral Maxillofac Surg 2009 47 4 323 4 10.1016/j.bjoms.2009.01.001 19243867

[B14] Vasconcelos BC Porto GG Lima FT Review Treatment of chronic mandibular dislocations using miniplates: follow-up of 8 cases and literature review Int J Oral Maxillofac Surg 2009 38 9 933 6 10.1016/j.ijom.2009.04.013 19467842

[B15] Caminiti MF Weinberg S Review Chronic mandibular dislocation: the role of non-surgical and surgical treatment J Can Dent Assoc 1998 64 7 484 91 9737079

[B16] Widmark G Surgical intervention in the temporomandibular joint Swed Dent J Suppl 1997 123 1 87 9352617

[B17] Miller GA Murphy EJ External pterygoid myotomy for recurrent mandibular dislocation. Review of the literature and report of a case Oral Surg Oral Med Oral Pathol 1976 42 6 705 16 10.1016/0030-4220(76)90091-8 1069214

[B18] Cardoso AB Vasconcelos BC Oliveira DM Comparative study of eminectomy and use of bone miniplate in the articular eminence for the treatment of recurrent temporomandibular joint dislocation Braz J Otorhinolaryngol 2005 71 1 32 7 16446889 10.1016/S1808-8694(15)31282-9 PMC9443489

[B19] Ueki K Marukawa K Nakagawa K Yamamoto E Condylar and temporomandibular joint disc positions after mandibular osteotomy for prognathism J Oral Maxillofac Surg 2002 60 12 1424 32 discussion 1432-4 10.1053/joms.2002.36098 12465004

[B20] Hu J Wang D Zou S Effects of mandibular setback on the temporomandibular joint: a comparison of oblique and sagittal split ramus osteotomy J Oral Maxillofac Surg 2000 58 4 375 80 10.1016/S0278-2391(00)90915-7 10759116

[B21] Guarda-Nardini L Palumbo B Manfredini D Ferronato G Surgical treatment of chronic temporomandibular joint dislocation: a case report Oral Maxillofac Surg 2008 12 1 43 6 10.1007/s10006-008-0097-5 18600361

[B22] Upton LG Sullivan SM The treatment of temporomandibular joint internal derangements using a modified open condylotomy: a preliminary report J Oral Maxillofac Surg 1991 49 6 578 83 10.1016/0278-2391(91)90338-M 2037913

[B23] Ghali GE Sikes JW Jr Intraoral vertical ramus osteotomy as the preferred treatment for mandibular prognathism J Oral Maxillofac Surg 2000 58 3 313 5 10.1016/S0278-2391(00)90063-6 10716115

[B24] Sindet-Pedersen S Intraoral myotomy of the lateral pterygoid muscle for treatment of recurrent dislocation of the mandibular condyle J Oral Maxillofac Surg 1988 46 6 445 9 10.1016/0278-2391(88)90409-0 3164048

[B25] Hall HD Werther JR Results of reoperation after failed modified condylotomy J Oral Maxillofac Surg 1997 55 11 1250 3 10.1016/S0278-2391(97)90178-6 9371115

[B26] Ohura N Ichioka S Sudo T Nakagawa M Kumaido K Nakatsuka T Dislocation of the bilateral mandibular condyle into the middle cranial fossa: review of the literature and clinical experience J Oral Maxillofac Surg 2006 64 7 1165 72 10.1016/j.joms.2006.03.043 16781355

[B27] Vasconcelos BC Porto GG Treatment of chronic mandibular dislocations: a comparison between eminectomy and miniplates J Oral Maxillofac Surg 2009 67 12 2599 604 10.1016/j.joms.2009.04.113 19925978

[B28] Shibata T Yamashita T Nakajima N Ueda M Ishijima T Shigezumi M Arisue M Treatment of habitual temporomandibular joint dislocation with miniplate eminoplasty: a report of nine cases J Oral Rehabil 2002 29 9 890 4 10.1046/j.1365-2842.2002.00900.x 12366587

[B29] Mangi Q Ridgway PF Ibrahim Z Evoy D Dislocation of the mandible Surg Endosc 2004 18 3 554 6 [Medline] 15114987 10.1007/s00464-003-4223-z

[B30] Lowery LE Beeson MS Lum KK The wrist pivot method, a novel technique for temporomandibular joint reduction J Emerg Med 2004 27 2 167 70 [Medline] 10.1016/j.jemermed.2004.03.007 15261360

[B31] Hoard MA Tadje JP Gampper TJ Edlich RF Traumatic chronic TMJ dislocation: report of an unusual case and discussion of management J Craniomaxillofac Trauma. Winter 1998 4 4 44 7 [Medline] 11951281

[B32] Ozcelik TB Pektas ZO Management of chronic unilateral temporomandibular joint dislocation with a mandibular guidance prosthesis: a clinical report J Prosthet Dent 2008 99 2 95 100 [Medline] 10.1016/S0022-3913(08)60024-4 18262009

[B33] Undt G Kermer C Piehslinger E Rasse M Treatment of recurrent mandibular dislocation, Part I: Leclerc blocking procedure Int J Oral Maxillofac Surg 1997 26 2 92 7 [Medline] 10.1016/S0901-5027(05)80634-4 9151160

[B34] Stone KC Humphries RL Maxillofacial and head trauma. Mandible fractures Current Diagnosis & Treatment Emergency Medicine 2008 6 McGraw Hill21681708

[B35] Harstall R Gratz KW Zwahlen RA Mandibular condyle dislocation into the middle cranial fossa: a case report and review of literature J Trauma 2005 59 6 1495 503 [Medline] 10.1097/01.ta.0000199241.49446.80 16394930

[B36] Schwab RA Genners K Robinson WA Clinical predictors of mandibular fractures Am J Emerg Med 1998 16 3 304 5 [Medline] 10.1016/S0735-6757(98)90108-5 9596439

[B37] Lee SH Son SI Park JH Park IS Nam JH Reduction of prolonged bilateral temporomandibular joint dislocation by midline mandibulotomy Int J Oral Maxillofac Surg 2006 35 11 1054 6 [Medline] 10.1016/j.ijom.2006.03.023 16829033

[B38] Ferretti C Bryant R Becker P Lawrence C Temporomandibular joint morphology following post-traumatic ankylosis in 26 patients Int J Oral Maxillofac Surg 2005 34 4 376 81 [Medline] 10.1016/j.ijom.2004.09.003 16053845

[B39] Talley RL Murphy GJ Smith SD Baylin MA Haden JL Standards for the history, examination, diagnosis, and treatment of temporomandibular disorders (TMD): a position paper American Academy of Head, Neck and Facial Pain. Cranio 1990 8 1 60 77 [Medline] 10.1080/08869634.1990.11678302 2098190

[B40] Luyk NH Larsen PE The diagnosis and treatment of the dislocated mandible Am J Emerg Med 1989 7 3 329 35 [Medline] 10.1016/0735-6757(89)90181-2 2653332

[B41] Schuknecht B Graetz K Radiologic assessment of maxillofacial, mandibular, and skull base trauma Eur Radiol 2005 15 3 560 8 [Medline] 10.1007/s00330-004-2631-7 15662492

[B42] Totten VY Zambito RF Propofol bolus facilitates reduction of luxed temporomandibular joints J Emerg Med 1998 16 3 467 70 [Medline] 10.1016/S0736-4679(98)00018-3 9610979

[B43] Chen YC Chen CT Lin CH Chen YR Ann Plast Surg 2007 58 1 105 8 [Medline] 10.1097/01.sap.0000232981.40497.32 17197953

[B44] Ardehali MM Kouhi A Meighani A Rad FM Emami H Temporomandibular joint dislocation reduction technique: a new external method vs. the traditional Ann Plast Surg 2009 63 2 176 8 [Medline] 10.1097/SAP.0b013e31818937aa 19542876

[B45] Shun TA Wai WT Chiu LC A case series of closed reduction for acute temporomandibular joint dislocation by a new approach Eur J Emerg Med 2006 13 2 72 5 [Medline] 10.1097/01.mej.0000192046.19977.5a 16525232

[B46] Bauss O Sadat-Khonsari R Fenske C Engelke W Schwestka-Polly R Temporomandibular joint dysfunction in Marfan syndrome Oral Surg Oral Med Oral Pathol Oral Radiol Endod 2004 97 5 592 8 [Medline] 10.1016/j.tripleo.2003.10.024 15153871

[B47] Chacon GE Dawson KH Myall RW Beirne OR A comparative study of 2 imaging techniques for the diagnosis of condylar fractures in children J Oral Maxillofac Surg 2003 61 6 668 72 discussion 673. [Medline] 10.1053/joms.2003.50134 12796873

[B48] Fonseca RJ Walker RV Management of injuries to the temporomandibular joint region Oral and Maxillofacial Trauma 1991 Philadelphia: WB Saunders Co 430 1

[B49] Gassner R Tuli T Hachl O Rudisch A Ulmer H Cranio-maxillofacial trauma: a 10 year review of 9,543 cases with 21,067 injuries J Craniomaxillofac Surg 2003 31 1 51 61 [Medline] 12553928 10.1016/s1010-5182(02)00168-3

[B50] Laskin DM Frederickson JM, Krause CJ Temporomandibular joint disorders 1993 Otolaryngology: Head and Neck Surgery. St. Louis: Mosby-Yearbook 1443 50

[B51] Thexton A A case of Ehlers-Danlos syndrome presenting with recurrent dislocation of the temporomandibular joint Br J Oral Surg 1965 3 190 3 [Medline] 14324132 10.1016/s0007-117x(64)80043-3

[B52] van der Linden WJ Dislocation of the mandibular condyle into the middle cranial fossa: report of a case with 5 year CT follow-up Int J Oral Maxillofac Surg 2003 32 2 215 8 Haddon R, Peacock IV WF. Face and Jaw Emergencies. Emergency Medicine: A Comprehensive Study Guide. 6th ed. McGraw Hill; 2004:1471-1476 10.1054/ijom.2002.0319 12729786

[B53] Mangi Q Ridgway PF Ibrahim Z Evoy D Dislocation of the mandible Surg Endosc 2004 18 3 554 6 [Medline] 15114987 10.1007/s00464-003-4223-z

[B54] Hoard MA Tadje JP Gampper TJ Edlich RF Traumatic chronic TMJ dislocation: report of an unusual case and discussion of management J Craniomaxillofac Trauma. Winter 1998 4 4 44 7 [Medline] 11951281

[B55] Stone KC Humphries RL Maxillofacial and head trauma. Mandible fractures Current Diagnosis & Treatment Emergency Medicine 2008 6 McGraw Hill21681708

[B56] Harstall R Gratz KW Zwahlen RA Mandibular condyle dislocation into the middle cranial fossa: a case report and review of literature J Trauma 2005 59 6 1495 503 [Medline] 10.1097/01.ta.0000199241.49446.80 16394930

[B57] Schwab RA Genners K Robinson WA Clinical predictors of mandibular fractures Am J Emerg Med 1998 16 3 304 5 [Medline] 10.1016/S0735-6757(98)90108-5 9596439

[B58] Ferretti C Bryant R Becker P Lawrence C Temporomandibular joint morphology following post-traumatic ankylosis in 26 patients Int J Oral Maxillofac Surg 2005 34 4 376 81 [Medline] 10.1016/j.ijom.2004.09.003 16053845

[B59] Tasanen A Lamberg MA Closed condylotomy in the treatment of recurrent dislocation of the mandibular condyle Int J Oral Surg 1978 7 1 1 6 10.1016/S0300-9785(78)80002-7 418012

[B60] Goret-Nicaise M Awn M Dhem A The morphological effects on the rat mandibular condyle of section of the lateral pterygoid muscle Eur J Orthod 1983 5 4 315 21 6581054 10.1093/ejo/5.4.315

[B61] Satoh K Suzuki H Matsuzaki S A type II lateral dislocation of bilateral intact mandibular condyles with a proposed new classification Plast Reconstr Surg 93 3 598 602 8115519

[B62] Chan TC Harrigan RA Ufberg J Vilke GM Mandibular reduction J Emerg Med 2008 34 4 435 40 [Medline] 10.1016/j.jemermed.2007.06.037 18242920

[B63] Nakashima M Yano H Akita S Tokunaga K Anraku K Tanaka K Traumatic unilateral temporomandibular joint dislocation overlooked for more than two decades J Craniofac Surg 2007 18 6 1466 70 [Medline] 10.1097/scs.0b013e31814fb5af 17993903

[B64] Menon S Sinha R Gap arthroplasty for mandibular condyle dislocation and impaction into the middle cranial fossa J Oral Maxillofac Surg 2008 66 11 2390 3 [Medline] 10.1016/j.joms.2008.01.064 18940513

[B65] Kato T Shimoyama T Nasu D Kaneko t Horie N Kubo I Autologous blood injection into articular cavity for the treatment of recurrent temporomandibular joint dislocation: a case report J Oral Sci 2007 49 237 9 10.2334/josnusd.49.237 17928731

[B66] Undt G Kermer C Rasse M Treatment of recurrent mandibular dislocation, Part II: Eminectomy Int J Oral Maxillofac Surg 1997 26 98 9151161 10.1016/s0901-5027(05)80825-2

[B67] Shorey CW Campbell JH Dislocation of the temporomandibular joint Oral Surg Oral Med Oral Pathol Oral Radiol Endod 2000 89 662 10.1067/moe.2000.106693 10846117

[B68] Whiteman PJ Pradel EC Bilateral temporomandibular joint dislocation in a 10-month-old infant after vomiting Pediatr Emerg Care 2000 16 418 10.1097/00006565-200012000-00011 11138886

[B69] Lovely FW Copeland RA Reduction eminoplasty for chronic recurrent luxation of the temporomandibular joint J Can Dent Assoc 1981 47 179 7013948

[B70] Kai S Kai H Nakayama E Clinical symptoms of open lock position of the condyle. Relation to anterior dislocation of the temporomandibular joint Oral Surg Oral Med Oral Pathol 1992 74 143 10.1016/0030-4220(92)90372-W 1508520

[B71] Awang MN A new approach to the reduction of acute dislocation of the temporomandibular joint: a report of three cases Br J Oral Maxillofac Surg 1987 25 244 10.1016/S0266-4356(87)80025-6 3474022

[B72] Leopard PJ Surgery of the non-ankylosed temporomandibular joint Br J Oral Maxillofac Surg 1987 25 138 10.1016/0266-4356(87)90008-8 3555613

[B73] Ziegler CM Haag C Muhling J Treatment of recurrent temporomandibular joint dislocation with intramuscular botulinum toxin injection Clin Oral Investig 2003 7 52 12673439 10.1007/s00784-002-0187-y

[B74] Güven O Management of chronic recurrent temporomandibular joint dislocations: a retrospective study J Craniomaxillofac Surg 2009 37 1 24 9 18996023 10.1016/j.jcms.2008.08.005

[B75] Güven O A clinical study on treatment of temporomandibular joint chronic recurrent dislocation by a modified eminoplasty technique J Craniofac surg 2008 19 5 1275 80 10.1097/SCS.0b013e31818437b3 18812851

[B76] Kuttenberger JJ Hardt N Long-term results following miniplate eminoplasty for the treatment of recurrent dislocation and habitual luxation of the temporomandibular joint Int J Oral Maxillofac Surg 2003 32 5 474 9 14759104

[B77] Debnath SC Kotrashetti SM Halli R Baliga S Bilateral vertical-oblique osteotomy of ramus (external approach) for treatment of a longstanding dislocation of the temporomandibular joint: A case report Oral Surg Oral Med Oral Pathol Oral Radiol Endod 2006 101 6 e79 82 10.1016/j.tripleo.2005.10.046 16731379

[B78] Costas López A Monje Gil F Fernandez Sanromán J Goizueta Adame C Castro Ruiz PC Glenohumeral osteotomy as a definitive treatment for recurrent dislocation of the jaw J Craniomaxillofac Surg 1996 24 3 178 83 8842910 10.1016/s1010-5182(96)80053-9

[B79] Sensŏz O Ustϋner Et Celebioğlu S Mutaf M Eminectomy for the the treatment of chronic subluxation and recurrent dislocation of the temporomandibular joint and a new method of patient evaluation Ann Plast Surg 1992 29 4 299 302 10.1097/00000637-199210000-00004 1466524

[B80] Bhandari S Swain M Dewoolkar LV Temporomandibular joint Dislocation after Laryngeal mask airway insertion The Internet Journal of Anaesthesiology 2008 16 1 1 4

[B81] Lipp M Von Domarus H Daublender M Temporomandibular joint dysfunction after endotracheal intubation Anaesthetisa 1987 36 442 45

[B82] Rastogi 'Nk Vakhari N Hung OR Perioperative anterior dislocation of the temporomandibular joint Anesth Analg 1997 84 924 26 9085984 10.1097/00000539-199704000-00042

[B83] Young AL Khan J Thomas Dc Quek SYP Use of Masseteric and Deep Temporal nerve blocks for reduction of mandibular dislocation Anesth Prog 2009 56 9 13 10.2344/0003-3006-56.1.9 19562887 PMC2662506

[B84] Lewis JES A simple technique for reduction of longstanding dislocation of mandible Br J Oral Surg 1981 19 52 56 10.1016/0007-117X(81)90021-4 6938241

[B85] Gambling DR Ross PL Temporomandibular joint subluxation on induction of anaesthesia (letter) Anesth and Analg 1988 3337354

[B86] Poswillo D Review surgery of the temporomandibular joint Oral Sci Rev 1974 6 87 118 4523952

[B87] MaCFarlane WI Recurrent dislocation of the mandible: treatment of seven cases by a simple lateral pterygoid myotomy Br J Oral 1977 14 227 9 10.1016/0007-117X(77)90029-4 265171

[B88] Thangarajah T McCulloch N Thangarajah S Stocker J Bilateral temporomandibular joint dislocation in a 29-year-old man: a case report J Med Case Reports 2010 4 263 10.1186/1752-1947-4-263 20698976 PMC2925850

[B89] Cascone P Ungari C Paparo F Marianetti TM Ramieri V Fatone M A new surgical approach for the treatment of chronic recurrent temporomandibular joint dislocation J Craniofac Surg 2008 19 2 510 2 10.1097/SCS.0b013e318163e42f 18362734

[B90] Daif ET Autolougous blood injection as a new treatment modality for chronic recurrent temporomandibular joint dislocation Oral Surg Oral Med Oral Pathol Oral Radiol Endod 2010 109 1 31 6 10.1016/j.tripleo.2009.08.002 19926503

[B91] Oliphant R Key B Dawson C Chung D Bilateral temporomandibular joint dislocation following pulmonary function testing: a case report and review of closed reduction techniques Emerg Med J 2008 25 435 436 10.1136/emj.2007.055038 18573960

[B92] Rosemore J Nikoomanesh P Lacy BE Bilateral temporomandibular joint dislocation after PEG tube placement Gasintest Endosc 2004 59 146 7 10.1016/S0016-5107(03)02362-9 14722573

[B93] Kepron W Bilateral dislocations of the temporomandidibular joint complicating fibreoptic bronchoscopy Chest 1986 90 465 3743170 10.1378/chest.90.3.465a

[B94] Schwartz AJ Dislocation of the mandible: a case report ANAA J 2000 68 507 13 11272957

[B95] Ting J Temporomandibular joint dislocation after use of laryngeal mask airway Anaesthesia 2006 61 201 16430591 10.1111/j.1365-2044.2005.04526.x

[B96] Avidan A Dislocation of the mandible due to forceful yawning during induction with propofol JClin Anaesth 2002 14 159 60 10.1016/S0952-8180(01)00374-9 11943534

[B97] Thomas ATS Wong TW Lau CC A case series of closed reduction for acute temporomandibular joint dislocation by a new approach Eur J Emerg Med 2006 13 72 5 10.1097/01.mej.0000192046.19977.5a 16525232

[B98] Hale RH Treatment of recurrent dislocation of the mandible: review of literature and report of cases J oral Surg 1972 30 527 30 4503606

[B99] Daelen B Thorwith V Koch A Treatment of recurrent dislocation of the temporomandibular joint with type A Botulinium toxin Int J Oral Maxillofac Surg 1997 26 458 60 10.1016/S0901-5027(97)80014-8 9418151

[B100] Laskin DM Myotomy for the management of recurrent and protracted mandibular dislocation Trans Int Conf Oral Surg 1973 4 264 268 4510527

[B101] Gould JF Shortening of the temporalis tendon for hypermobility of the temporomandibular joint J Oral Surg 1978 36 781 3 280646

[B102] Puelacher WC Waldhart E Miniplate Eminoplasty: a new surgical treatment for TMJ dislocation J Craniomaxillofac Surg 1993 2 176 8 10.1016/s1010-5182(05)80109-x 8335731

[B103] Shakya S Ongole R Sumanth KN Denny CE Chronic bilateral dislocation of temporomandibular joint Kathmandu University Medical Journal 2010 8 251 256 10.3126/kumj.v8i2.3570 21209547

[B104] Myrhaug H A new method of operation for Habitual dislocation of the mandible: review of former methods of treatment Acta Odontol Scand 1951 9 247 61 10.3109/00016355109012789 14877536

[B105] Pogrel MA Articular Eminectomy for recurrent dislocation Br J Oral Maxillofac Surg 1987 25 237 43 10.1016/S0266-4356(87)80024-4 3474021

[B106] Irby WB Surgical Correction of chronic dislocation of the temporomandibular joint not responsive to conservative therapy J Oral Surg 1957 15 307 12 13476292

[B107] Oatis GW Jr Baker DA The bilateral eminectomy as definitive treatment. A review of 44 patients Int J Oral Surg 1984 13 294 8 10.1016/S0300-9785(84)80036-8 6434448

[B108] Helman J Laufer D Minkov B Gutman D Eminectomy as surgical treatment for chronic mandibular dislocations Int J Oral Surg 1984 47 179 84 10.1016/s0300-9785(84)80018-6 6439656

[B109] Magge SN Chen HI Carrasco LR Storm PB Dislocation of mandible into the middle cranial fossa-a case report J Neurosurg 2007 107 75 78 17644926 10.3171/PED-07/07/075

[B110] Avrahami E Rabin A Mejdar SI Medial dislocation of the temporomandibular joint Neuroradiology 1997 39 602 604 10.1007/s002340050476 9272501

[B111] Rattan V Arora S Prolonged temporomandibular joint dislocation in an unconscious patient after airway manipulation Anaesth Analg 2006 102 1294 10.1213/01.ANE.0000199217.90585.B5 16551954

[B112] Adekeye EO Inverted L shaped osteotomy for prolonged bilateral dislocation of the temporomandibular joint J oral Surg 1976 41 563 576 10.1016/0030-4220(76)90308-x 1063959

[B113] Marx RE Garg AK Dental and Craniofacial Applications of Platelet-Rich Plasma 2005 Qiuntessence publishing. Chicago

[B114] Karabouta I Increasing the articular eminence by the use of blocks of porous coralline hydroxyapatite for treatment of recurrent joint doislocation J Craniomaxillofac surg 1990 18 107 9 2160986 10.1016/s1010-5182(05)80325-7

[B115] Lawlor MG Recurrent dislocation of the mandible: treatment of ten cases by Dautery procedure Br J Oral Surg 1982 20 14 21 10.1016/0007-117X(82)90002-6 6950786

[B116] Tesfaya Y Skorzewska A lai S Hazard of yawning CMAJ 1991 14 156 PMC1336069 1742691

